# Decolonising Indigenous Health Research Requires Redefining Sex, Sexuality and Gender

**DOI:** 10.5694/mja2.70320

**Published:** 2026-10-16

**Authors:** Shakara Liddelow‐Hunt, James H. L. Hill, Kate Daglas, Kaiwarr Clancy, Madi Day, Alistair Ott

**Affiliations:** ^1^ Australian Research Centre in Sex, Health & Society La Trobe University Melbourne Victoria Australia; ^2^ Youth Mental Health The Kids Research Institute Australia Nedlands Western Australia Australia; ^3^ The University of Western Australia School of Psychological Science Perth Western Australia Australia; ^4^ Shifting Ground Broome Western Australia Australia; ^5^ Poche Centre for Indigenous Health The University of Sydney, Faculty of Medicine and Health Sydney New South Wales Australia; ^6^ Faculty of Social Sciences Western Sydney University Sydney New South Wales Australia; ^7^ College of Arts and Social Science Australian National University Canberra Australian Capital Territory Australia

**Keywords:** data collection, gender identity, sexuality, social justice

## Abstract

LGBTQA+SB (lesbian, gay, bisexual, transgender, intersex, queer, asexual, Sistergirl and Brotherboy) participants are rarely identified in Aboriginal and Torres Strait Islander health research data, resulting in persistent gaps in the literature. In this article, we briefly explore the implications of LGBTQA+SB erasure in Aboriginal and Torres Strait Islander health research for decolonisation, policy and practice. We outline approaches to collecting quantitative data, examples of Indigenous research that did identify LGBTQA+SB participants and potential challenges for data collection. Ultimately, we argue that decolonisation requires re‐thinking how we define sex, sexuality and gender, unsettling Western biomedical norms that are pervasive in health research.

## 1 Introduction

In 2015, an Aboriginal and Torres Strait Islander Suicide Prevention Evaluation Project (ATSISPEP) roundtable identified the need for mental health statistics concerning Aboriginal and Torres Strait Islander LGBTQA+SB (lesbian, gay, bisexual, transgender, intersex, queer, asexual, Sistergirl and Brotherboy) people [1], reflecting a wider call from community advocates [2]. In 2023, the first national statistics were self‐published from the Walkern Katatdjin national survey revealing that nearly half (45.4%) of Aboriginal and Torres Strait Islander LGBTQA+ young people in the study had attempted suicide in their lifetime [3]. Despite limitations to this survey's generalisability, it is worth contextualising against a similar survey in south‐east Queensland during the same period, which indicated that 21% of Aboriginal and Torres Strait Islander adults had attempted suicide [4]. Further, 5.3% of randomly sampled adolescents aged 16–17 years who completed the Australian Child and Adolescent Survey of Mental Health and Wellbeing survey in 2015 had attempted suicide [5]. Delays between advocacy and research are not uncommon, but given the investment into Aboriginal and Torres Strait Islander suicide prevention in recent decades, it is concerning that a substantial proportion of the Aboriginal and Torres Strait Islander population [6, 7] with notably higher rates of suicidality are largely absent from the Aboriginal and Torres Strait Islander mental health literature.

In 2026, it is still uncommon for Aboriginal and Torres Strait Islander health research of any kind to collect and report accurate data about the sex, gender and sexuality of participants. Walter critiques the overabundance of Indigenous statistics that are decontextualised, simplistic, homogenising and unresponsive to community priorities [8]. Extending this, we would argue that the erasure of bodily, sexual and gender diversity within Indigenous data is an artefact of pervasive norms concerning gender and sexuality that originate in colonialism [9].

## 2 Personhood and the Colony

Colonisation has negatively impacted ways of life for all Aboriginal and Torres Strait Islander peoples, including the ways in which understandings of gender, sexuality, sex, bodies, and family and kinship systems have been restricted, denied and intentionally erased by the colonial system [9]. It is now difficult to locate queerness in our histories as a direct result of systemic violence that has targeted and erased diversity [10]. Despite this, LGBTQA+SB Aboriginal and Torres Strait Islander people continue to share stories, perspectives and lived experiences outside a colonial cisheteronormative lens [11]. In the 21st century, colonial influence manifests in data collection through adherence to a medical norm of male/female bodies, even where exploring culturally defined gender roles and bodily diversity is warranted. Alternative approaches towards sex, sexuality and gender may include defining romantic relationships by their accordance with kinship systems, understanding gender via participation in cultural practices or centring connections between body, spirit and Country.

## 3 Illustrative Example

The 2021 National Community Attitudes towards Violence against Women Survey utilised the Australia Bureau of Statistics (ABS) Standard for Sex, Gender, Variations of Sex Characteristics and Sexual Orientation Variables 2020 (henceforth ‘the ABS Standard’) [12], alongside identifying whether participants were Aboriginal and/or Torres Strait Islander [13]. All Aboriginal and Torres Strait Islander participants' data were later disaggregated and reported, and LGBTQ+ identity was included as a covariate in the analyses. Findings showed that 11% of Aboriginal and Torres Strait Islander participants were sexuality diverse, LGBTQ+ participants were less likely to trust police to respond appropriately to family violence, and all participants were less likely to recognise an LGBTQA+SB‐specific form of coercive control as family violence [13]. These findings are indicative of specific inequities in police and community responses to family violence, with real implications for prevention and intervention that would have otherwise been missed.

## 4 Potential Challenges

Currently, no clear recommendations exist for collecting data regarding sex, sexuality and gender in Aboriginal and Torres Strait Islander health research. In Australia, quantitative researchers are advised to follow the ABS Standard, which consists of two multiple‐choice items that differentiate between gender and sex presumed at birth, one item for sexuality and one item concerning innate variations of sex characteristics. The ABS Standard produces reliable data when used with the Australian general population; however, it was not developed with Aboriginal and Torres Strait Islander communities, nor is the ABS a leader in decolonising data and research [8].

The terms ‘Sistergirl’ and ‘Brotherboy’ may be adopted as gender options, given that they are increasingly used by LGBTQA+SB communities to acknowledge uniquely Aboriginal and Torres Strait Islander concepts of gender diversity. The terms are effective in signalling inclusivity, but their efficacy in non‐LGBTQA+SB‐specific research is dubious. People are more likely to identify as Sistergirl or Brotherboy based on their participation in local communities, and very few Aboriginal and Torres Strait Islander young people self‐identify as Sistergirl or Brotherboy as of 2022 [3].

The relative size of the Aboriginal and Torres Strait Islander LGBTQA+SB population results in small sample sizes in research that necessarily constrain the types of research and analysis that are possible even when data are collected. In clinical research and health service delivery, practitioners may be hesitant to discuss sexuality and gender diversity due to perceived heteronormativity within the Aboriginal and Torres Strait Islander community. Additionally, LGBTQA+SB people may be hesitant to disclose their gender or sexuality due to ongoing discrimination [14].

## 5 Ways Forward

Improvements to LGBTQA+SB data collection will not occur through prompt development of a perfect measure, but through a long‐term iterative process of community education and discussion. For example, sharing experiences of diversity with Elders creates space to explore the defining features of genders and romantic relationships within each culture. Additionally, collecting accurate data relies on research participants feeling safe to disclose that they are LGBTQA+SB, which will follow from slow cultural shifts and transparent communication of a commitment to inclusivity.

Other terms for genders, bodies and attraction, including words borrowed from Aboriginal and Torres Strait Islander languages, may be appropriate for use in localised studies. Internationally, terms such as Two‐Spirit (North America) and Takatāpui (Māori) are used flexibly across sex, sexuality and gender because they are defined by Indigenous cultural roles [15, 16]. For example, participants in the Youth19 survey of high school students in Aotearoa had the option of selecting Takatāpui for both gender and sexuality, which later informed intersectional analyses of their well‐being [15].

Quantitative research should not fear reporting on Aboriginal and Torres Strait Islander LGBTQ+SB people within their research due to small sample sizes, provided that data are properly deidentified. This is particularly true for even smaller populations within the Aboriginal and Torres Strait Islander LGBTQA+SB community, such as Sistergirls and Brotherboys. Thoughtful consideration should be given to the respective limitations and ethics of conducting analysis with low statistical power versus excluding under‐served populations [17]. Qualitative and mixed‐methods research can be drawn on to interrogate the influence of sex, sexuality and gender on health outcomes and behaviours when it is not possible to determine this from quantitative research alone.

The collection of gender, sex and sexuality data is not solely the responsibility of individual researchers, but related to institutional policy and community partnerships. We welcome the National Health and Medical Research Council's (NHMRC) position that all NHMRC‐funded research should utilise the ABS Standard or other relevant variables [18]. However, the way forward is also dependent on Aboriginal and Torres Strait Islander LGBTQA+SB leadership and research partnerships to navigate the challenges described in this article.

## 6 Lived Realities (Positionality)

The authors are a queer Yamatji person, a Wiradjuri trans fulla, a Wakka Wakka Brotherboy, a trans Ngarrindjeri man, a queer Gunditjmara woman and a trans Murri living and working across Noongar, Wurundjeri, Ngunnawal Ngambri, Dharug and Wiradjuri Country. The author team have arrived at this article via our involvement in a National Aboriginal and Torres Strait Islander LGBTQA+SB Research Governance Group. The core mission of the group is to advance Indigenous Data Sovereignty for Aboriginal and Torres Strait Islander LGBTQA+SB peoples. This necessitates, first, that these data exist. Individually, we have all navigated the silence concerning our lived reality in both our personal and professional lives. From these experiences, we know that we need data about our Aboriginal and Torres Strait Islander LGBTQA+SB communities if we are to champion our own rights [8, 19]. The CONSIDER reporting criteria checklist for health research involving Indigenous peoples found in the Supporting Information provides further details of our collective engagement [19].

As of 2024, LGBTQA+SB people are now mentioned as a population requiring special consideration in the *National Aboriginal and Torres Strait Islander Suicide Prevention Plan* [20], following from the findings of the ATSISPEP roundtable [1]. The evidence base concerning Aboriginal and Torres Strait Islander LGBTQA+SB mental health is gradually growing but it is not yet sufficient to meet community demand, and there are a considerable number of health topics for which no evidence about Aboriginal and Torres Strait Islander LGBTQA+SB people exists. Aboriginal and Torres Strait Islander LGBTQA+SB contend with additional stressors and barriers to accessing health care [3], possibly resulting in higher rates of other preventable disease, but nothing is currently known. The shortage of evidence perpetuates ongoing inequities via gaps in funding allocation, practice guidelines and health policy. Research with Aboriginal and Torres Strait Islander LGBTQA+SB people may not yet be easy, but in this context, we have to wonder: Is it really that hard?

## 7 Conclusion

We argue for the necessity of re‐thinking sex, sexuality and gender diversity in Indigenous health research from our own perspectives as LGBTQA+SB people, but these issues extend to the rest of our Aboriginal and Torres Strait Islander communities. If we are to progress towards a decolonised understanding of health for all Aboriginal and Torres Strait Islander people, we must uproot the discourses that frame our bodies, relationships, gender roles, family structures and connection to Country within a biomedical tradition that has rarely acknowledged our humanity.

## Author Contributions

Shakara Liddelow‐Hunt: conceptualisation, project administration, writing – original draft. James H.L. Hill: conceptualisation, writing – review and editing. Kate Daglas: conceptualisation, writing – review and editing. Kaiwarr Clancy: conceptualisation. Madi Day: conceptualisation. Alistair Ott: conceptualisation, writing – original draft.

## Funding

The authors have nothing to report.

## Disclosure

Not commissioned; externally peer reviewed. No AI was used in the development of this manuscript.

## Conflicts of Interest

The authors declare no conflicts of interest.

## Supporting information


**Data S1:** CONSIDER Statement.

## Data Availability

This article includes no original data.
