# Supplementary material for: Decolonising Indigenous Health Research Requires Redefining Sex, Sexuality and Gender
Source: Med J Aust. 2026 Oct 16;224(10):e70320. doi: 10.5694/mja2.70320 (PMC13673996; doi:10.5694/mja2.70320)
Supplement: Supplementary file 1 [file MJA2-224-0-s001.pdf]

MJA
The Medical Journal of Australia

Supporting Information

Supplementary material

**This appendix was part of the submitted manuscript and has been peer reviewed.
It is posted as supplied by the authors.**

Appendix to: Liddelow-Hunt S, Hill JHL, Daglas K, et al. Decolonising Indigenous health research requires redefining sex, sexuality and gender. *Med J Aust* 2026; doi: 10.5694/mja2.70320.

How the authors have addressed the CONSIDER checklist[1] of items to include when reporting health research involving Indigenous Peoples

[1] Huria T, Palmer SC, Pitama S, et al. Consolidated criteria for strengthening reporting of health research involving indigenous peoples: the CONSIDER statement. BMC Medical Research Methodology. 2019;19(1):173. doi:10.1186/s12874-019-0815-8

**Governance**
This manuscript was generated as part of the activities of a standing National Aboriginal and Torres Strait Islander LGBTQASB+ Research Governance Group. The Governance Group is collective of Aboriginal and Torres Strait Islander LGBTQA+SB people from across Australia, who have come together to guide the research needed to support their communities. This responds to an urgent need for strengths-based research about the wellbeing of Aboriginal and Torres Strait Islander LGBTQA+SB people, with continued community oversight over all stages of research. The Governance Group aims to enable research that results in meaningful benefit to Aboriginal and Torres Strait Islander LGBTQAB+ communities. By forming one continuing group with oversight across several research projects, the Governance Group hopes to facilitate future planning, collaboration, translation, and capacity building. This model is intended to more meaningfully enact the Maiam nayri Wingara principles for Indigenous Data Sovereignty, as compared to project-based research governance and partnerships in which the researchers initiating and facilitating the engagement retain greater power.<br><br>The Governance Group operates according to collectively authored Terms of Reference. Fundamental principles within the Terms of Reference include respect and care towards each, action driven by reciprocal obligations to each member's wider community, consensus decision-making, recognition of diversity, and a proactive approach to conceptualising and advocating for future research. In addition, the Terms of Reference requires that all members retain their Intellectual Property (IP), including Indigenous Cultural and Intellectual Property (ICIP), and no IP or ICIP will be shared outside of the Governance Group without permission. Governance Group members are reimbursed for their participation in Governance Group meetings, except where participation overlaps with existing professional commitments.<br><br>This manuscript was approved by the Governance Group, meaning that members who are *not* authors of the manuscript have also provided endorsement. As such, the authors are accountable to the wider Governance Group.
**Prioritization**
The idea for the manuscript emerged in response to all Governance Group member's experiences advocating for LGBTQA+SB inclusion in Aboriginal and Torres Strait Islander health and wellbeing settings. This includes specific advocacy regarding the identification of Aboriginal and Torres Strait Islander LGBTQA+SB people in research. The perspectives held by each Governance Group member are further informed by priorities within their local community, experiences working with and within Aboriginal Community Controlled Organisations, and awareness of the need for evidence to support policy change and funding allocation.
**Relationships (Indigenous stakeholders/participants and Research Team**
This manuscript does not report original research. As such, no ethical approval was undertaken. This manuscript was informed by the Maiam nayri Wingara principles for Indigenous Data Sovereignty, and similar international principles (e.g., OCAP).<br>All authors are Aboriginal and Torres Strait Islander LGBTQA+SB people who are members of a Governance Group, as described above. As such, all aspects of manuscript preparation were entirely Indigenous-led.<br>The first author, Shakara Liddelow-Hunt, is a Yamatji Research Fellow who holds a PhD in Population and Global Health. They have conducted research concerning Aboriginal and Torres Strait Islander LGBTQA+SB social and emotional wellbeing since 2019.<br>Alistair Ott is a Wiradjuri currently completing a PhD concerning blak trans joy in the college of Arts and Social Sciences. They have significant experience in peer work with trans and gender diverse communities.<br>James Hill is a Ngarrindjeri clinical psychology registrar currently completing a PhD in Psychology focused on anticipated stigma among trans and gender diverse youth.<br>Kate Daglas is a Gunditjmara currently working in mental health. They bring further expertise in training, Aboriginal methodologies, and criminology.<br>Kaiwarr Clancy is a Wakka Wakka Research Assistant at the University of Sydney, with extensive experience in research concerning Aboriginal and Torres Strait wellbeing, including current research on diabetes and alcohol and other drugs.<br>Madi Day is a Murri Associate Professor at the University of Western Sydney, with a PhD in Indigenous Studies. They have led multiple research projects concerning Aboriginal and Torres Strait Islander LGBTQA+SB, including works for the Australian Institute of Health and Welfare.
**Methodologies**
This perspective article does not report on original research.

**Participation**
This perspective article does not report on original research.
**Capacity**
This article contributes to the publication record of the authors, who are all Aboriginal people. Additionally, participation in the National Aboriginal and Torres Strait Islander LGBTQA+SB Governance Group contributes to the capacity of members through: a) ongoing learning sessions, b) opportunities to contribute to research projects governed by the group as co-analysts and/or co-authors on publications, c) Indigenous Data Sovereignty and Indigenous Data Governance experience. Beyond these formal mechanisms, members benefit from their connections with each other and mutual mentorship and collaboration.
**Analysis and interpretation**
This perspective article does not report on original research.
**Dissemination**
This perspective article does not report on original research. The challenges and ways forward shared in this manuscript will continue to be shared and enacted, through the Governance Group's role in engaging with research projects. Additionally, members of the group will continue to advocate for the ways forward in their individual careers, working relationships with relevant stakeholders, and via conference presentations.
